# Impact of Financial Burden on Quality of Life of Caregivers for Cancer: The Moderating Role of Employment Status

**DOI:** 10.1093/geroni/igaf122.3204

**Published:** 2025-12-31

**Authors:** Khunza Asma

**Affiliations:** University of South Florida, Tampa, Florida, United States

## Abstract

Caregiving for cancer patients imposes substantial financial burdens on caregivers, which can adversely affect their quality of life. This study investigates the relationship between the financial burden of caregiving for cancer patients and the quality of life of caregivers, with a focus on the moderating role of employment status. Using data from the HRS 2020 dataset, we conducted a moderation analysis to explore how employment status influences this relationship. The independent variables in this study are income and wealth, representing the financial burden, while the dependent variable is the quality of life, measured by life satisfaction. Employment status serves as the moderating variable. Our findings reveal that financial burden significantly impacts the quality of life of caregivers. Caregivers with higher financial burdens report lower quality of life. However, employment status plays a crucial moderating role in this relationship. Employed caregivers report higher quality of life compared to their unemployed counterparts, even when experiencing similar levels of financial burden. This suggests that employment provides not only financial resources but also social support and a sense of purpose, which can mitigate the negative effects of financial strain. The results underscore the importance of policies that support employment opportunities for caregivers and provide financial assistance to those who are unemployed. By addressing the financial challenges faced by caregivers, we can improve their overall well-being and ability to provide care. These findings have significant implications for policymakers, healthcare providers, and support organizations aiming to enhance the well-being of caregivers.

